# Accumulation of embryos to improve outcomes in advanced-age women undergoing IVF/ICSI cycles: a retrospective cohort study

**DOI:** 10.3389/fendo.2026.1859978

**Published:** 2026-07-07

**Authors:** Mingya Cao, Yue Wang, Liang Zhou, Kexin Xing, Huanjun Li, Yuanyuan Liu, Qingyun Sun, Xuli Zhu, Zhiming Zhao

**Affiliations:** 1 Department of Reproductive Medicine, The Second Hospital of Hebei Medical University, Shijiazhuang, China; 2 Department of Reproductive Medicine, Central Hospital of Qinghe County, Xingtai, China

**Keywords:** accumulation of embryos, accumulation of oocytes, advanced maternal age, cumulative live birth rate, IVF/ICSI

## Abstract

**Objective:**

To investigate the effect of the embryo accumulation strategy prior to embryo transfer on reproductive outcomes among advanced-age women undergoing IVF/ICSI treatment.

**Methods:**

This retrospective study included 970 advanced-age female patients undergoing *in vitro* fertilization/intracytoplasmic sperm injection (IVF/ICSI) treatment at the Second Hospital of Hebei Medical University from 2012 to 2022. Participants were stratified into two groups according to the implementation of the embryo accumulation strategy before embryo transfer. The embryo accumulation group comprised 325 patients who completed ≥2 consecutive oocyte retrieval cycles and yielded at least one transferable embryo. The control group included 645 patients who underwent a single oocyte retrieval cycle and received embryo transfer with available transferable embryos. To eliminate baseline confounding bias, 1:1 propensity score matching (PSM) was applied. Ultimately, 299 matched pairs (598 patients) were successfully matched. Baseline clinical characteristics, laboratory indicators, and pregnancy outcomes were compared between the two cohorts before and after PSM. Multivariable logistic regression models were constructed to assess the independent effect of embryo accumulation on pregnancy outcomes. Restricted cubic spline and threshold effect analyses were performed to explore the nonlinear association between cumulative embryo number and reproductive outcomes. The primary study endpoint was cumulative live birth rate (CLBR), and the secondary endpoint was cumulative clinical pregnancy rate (CCPR).

**Results:**

After PSM, the biochemical pregnancy rate, CLBR and CCPR in the embryo accumulation group were all significantly higher than those in the control group (P < 0.001). However, no statistically significant difference was observed in the miscarriage rate between the two groups (P = 0.583). Binary logistic regression analysis based on the fully adjusted model (Adjust II) demonstrated that embryo accumulation was correlated with increased CLBR (OR = 2.75, 95% CI: 1.87–4.05, P < 0.001) and CCPR (OR = 2.71, 95% CI: 1.87–3.92, P < 0.001). Smooth curve fitting and threshold effect analysis were performed within the embryo accumulation group. CLBR and CCPR increased with the rise in cumulative embryo number when the cumulative embryo number was ≤ 2 (OR = 3.11, 95% CI: 1.27–8.50, P = 0.0181; OR = 3.59, 95% CI: 1.47–9.74, P = 0.0073). When the cumulative embryo number was > 2, CLBR and CCPR still presented a mild upward trend, but such trends did not reach statistical significance (OR = 1.06, 95% CI: 0.91–1.23, P = 0.4216; OR = 1.11, 95% CI: 0.97–1.29, P = 0.1326).

**Conclusions:**

Among advanced-age women receiving IVF/ICSI treatment, embryo accumulation via consecutive stimulation cycles can significantly improve CLBR and CCPR, and the clinical benefits are prominent when the number of accumulated embryos does not exceed two. This strategy effectively optimizes reproductive outcomes for advanced patients undergoing assisted reproductive technology (ART). Nevertheless, due to the limitations of this retrospective study and unadjusted confounding factors, embryo quantity should not be the sole basis for clinical decisions. Individualized treatment strategies shall be developed based on a comprehensive assessment of patients’ age, physical status and economic conditions.

## Introduction

Reproductive potential in women is strongly correlated with age, with a substantial decrease in natural conception rates beginning after the age of 35 (1). Women over 40 are in the late stage of their reproductive lifespan, and even with assisted reproductive technologies such as *in vitro* fertilization and embryo transfer (IVF-ET), clinical pregnancy and live birth rates are substantially lower. By age 45, the pregnancy rate drops to approximately 10% (2). With the continuous advancement and widespread application of ART, there has been a significant global increase in the number of advanced-age women seeking ART for fertility assistance. However, women with DOR experience a reduction in both oocyte quantity and quality, which yields fewer transferable embryos during IVF cycles. This outcome consequently diminishes clinical pregnancy and live birth rates. Optimizing fertility outcomes for women of advanced maternal age therefore remains a persistent clinical challenge.

To improve treatment efficacy in this patient population, numerous strategies have been proposed. Nevertheless, to date, no single intervention or adjustment in treatment plans has been conclusively demonstrated to yield definitive therapeutic benefits (3–5). Previous studies have demonstrated that increasing gonadotropin dosage does not improve the number of oocytes/embryos or pregnancy rates in predicted poor responders during a single IVF cycle (6–9). However, accumulating oocytes or embryos through consecutive stimulation cycles may represent a novel strategy to improve the adverse outcomes of poor ovarian response (POR) (10–12). Chatziparasidou A et al (10). proposed that repeated ovarian stimulation combined with vitrification of accumulated oocytes or embryos could provide poor responders undergoing preimplantation genetic testing (PGT) with more opportunities for high-quality embryo selection and multiple embryo transfers, thereby increasing the likelihood of a healthy pregnancy. According to data reported by the Cobo A team, the strategy of accumulating oocytes to obtain a larger cohort of oocytes/embryos significantly reduces patient dropout rates and cycle cancellation rates while effectively improving the cumulative success rate of frozen-thawed embryo transfers using surplus cryopreserved embryos (12). Ubaldi F et al. (13) demonstrated that transferring embryos derived from both fresh and vitrified oocytes yields higher cumulative sustained pregnancy rates. Greco E et al. (14) proposed that accumulating vitrified oocytes in modified natural cycles to increase embryo numbers, followed by their concurrent transfer with embryos developed in the preceding fresh cycle, can significantly enhance clinical pregnancy rates. Nevertheless, these studies lack consensus regarding the optimal accumulation methodology, the number of consecutive stimulation cycles, or the target number of oocytes and embryos to be accumulated.

A recent study investigating the correlation between oocyte yield and CLBR reported (15) that in advanced-age patients (≥38 years), a continued increase in predicted CLBR was observed beyond 15 oocytes retrieved, rising from 41.3% to 53.4% to 58.7% for 15-19, 20-24, and ≥25 oocytes, respectively. For advanced-age women, the adverse pregnancy outcomes of a single IVF/ICSI cycle are typically attributed to high cycle cancellation rates or the absence of high-quality embryos available for transfer. DOR in advanced-age women limits the number of oocytes or embryos obtainable within a single stimulation cycle. Therefore, accumulating a sufficient quantity of oocytes and embryos through multiple consecutive stimulation cycles may serve as a promising strategy to increase the number of embryo transfers or transfer attempts, thereby improving cumulative pregnancy rates and live birth rates. This approach could potentially enhance the poor IVF/ICSI outcomes observed in this population. Current research on cumulative pregnancy rates primarily focuses on improving pregnancy outcomes in POR patients through oocyte cryopreservation, whereas studies investigating the impact of cumulative embryos on pregnancy outcomes in advanced-age women remain limited. Although oocyte cryopreservation techniques have achieved considerable maturity, oocytes remain more fragile compared to embryos. For women with diminished ovarian reserve, cryopreserving embryos rather than oocytes appears to be a more judicious choice for both clinicians and patients. However, a recent study by Chen Zijiang et al. (16) suggested that FET is associated with poorer outcomes compared to fresh embryo transfer in patients with a poor prognosis. This may be attributed to potential embryo damage during the cryopreservation process. Nevertheless, the study also acknowledged limitations, such as differences in the number of embryos transferred, embryo developmental stage, and protocols for frozen-thawed cycles, which may affect the generalizability of the conclusions. Additionally, their study compared the live birth rate (LBR) per single thaw cycle with that of fresh cycles. It is noteworthy that both poor-prognosis and advanced-age populations are typically characterized by limited oocyte yield and embryo number per cycle, restricting the selection of available embryos for transfer. Therefore, rather than focusing on the LBR per single cycle, CLBR may be a more clinically relevant outcome measure for these patient groups. Datta AK et al. (17) recently reported in a study that performing embryo transfer after accumulating embryos over three IVF cycles could improve the live birth rate and reduce the risk of having no embryos available for transfer in low responders aged ≥35 years, thereby improving pregnancy outcomes in patients with POR. However, the study only mentioned the number of consecutive stimulation cycles without addressing the number of accumulated embryos. Therefore, several questions remain worthy of investigation, such as whether the strategy of accumulating embryos before transfer improves clinical outcomes in advanced-age women, whether cryopreservation-related embryo damage affects transplantation efficacy, what is the relationship between the number of accumulated embryos and pregnancy outcomes, and whether an increased number of embryos can compensate for the differences between frozen and fresh embryo transfer cycles.

In summary, this retrospective study compared pregnancy outcomes between cumulative embryo transfer and non-cumulative embryo transfer in advanced-age infertile women undergoing IVF/ICSI. Furthermore, it analyzed the correlation between the number of cumulative embryos and both the CLBR and CCPR. The study aimed to explore the clinical significance of cumulative embryo transfer and the optimal number of cumulative embryos for improving pregnancy outcomes in this population, thereby providing scientific evidence and data support for optimizing ART treatment strategies for advanced-age women.

## Materials and methods

### Study population and design

Patients undergoing IVF/ICSI treatment at the Reproductive Medicine Center of the Second Hospital of Hebei Medical University between January 2012 and December 2022 were enrolled, with female age ≥ 35 years defined as the inclusion criterion. The exclusion criteria were as follows: ① cycles involving oocyte cryopreservation and thawing; ② oocyte donation or donor sperm cycles; ③ patients with recurrent spontaneous abortion or comorbidities affecting pregnancy outcomes; ④ preimplantation genetic testing (PGT) cycles; ⑤ cases lost to follow-up with missing clinical outcome data; ⑥ cycles with no oocytes retrieved in the initial attempt. All relevant data were retrieved from the center, and this study was conducted as a retrospective cohort analysis. Propensity score matching was adopted to reduce baseline confounding bias for subsequent intergroup comparisons. Binary logistic regression was used to evaluate the association between cumulative embryo number and pregnancy outcomes. Smooth curve fitting and threshold effect analysis were performed to explore the relationships between cumulative embryo number and cumulative live birth rate and cumulative clinical pregnancy rate, and to identify the optimal cut-off value for cumulative embryos.

### Protocols and embryo culture

#### Ovarian stimulation protocols

The ovarian stimulation protocols were performed according to the standard procedures of our center. The primary protocols included the long agonist protocol, antagonist protocol, and ultra-long protocol, with supplementary individualized protocols such as the minimal stimulation protocol, luteal phase stimulation protocol, and natural cycle oocyte retrieval when clinically indicated. During controlled ovarian stimulation (COS), follicular diameter was monitored via transvaginal ultrasound, and serum levels of luteinizing hormone (LH), estradiol (E_2_), and progesterone (P) were measured. When two to three dominant follicles exceeding 18 mm in diameter were observed, a trigger dose of 10, 000 IU recombinant human chorionic gonadotropin (hcg) was administered. Oocyte retrieval was performed 36–38 hours later. The retrieved oocytes were fertilized via IVF or ICSI. Pronuclear (PN) evaluation was conducted 16–18 hours post-fertilization. The fertilized oocytes were cultured in G1-plus medium (Gothenburg, Sweden) until day 3 embryos. In the control group, one or two high-quality embryos were selected for either fresh embryo transfer or cryopreservation followed by frozen-thawed embryo transfer in subsequent cycles. In the cumulative embryo group, all embryos were cryopreserved after completing ≥2 consecutive oocyte retrieval cycles, followed by frozen-thawed embryo transfer in subsequent cycles.

Available embryos were defined by uniform criteria in this study. Embryos with abnormal fertilization (≥3 pronuclei, 3PN) were excluded from the available embryo pool. Day 3 cleavage-stage embryos were considered available when they possessed ≥5 blastomeres with a fragmentation rate lower than 25%. Day 5–6 blastocysts were classified as available if they reached an expansion grade of ≥3, with either the inner cell mass or trophectoderm graded B or higher.

High-quality embryos were screened according to strict morphological standards. Qualified day 3 cleavage-stage high-quality embryos contained 7 to 9 blastomeres with a fragmentation rate below 10% and unevenly sized blastomeres affecting less than half of all embryonic cells. High-quality blastocysts required no less than 3 expansion grades, with both the inner cell mass and the trophectoderm achieving grade B or higher morphology.

#### Embryo freezing and thawing

Embryo cryopreservation was performed using the vitrification method with commercially available cryopreservation kits (Kitazato, BioPharma Co., Japan) and warming kits (Kitazato, BioPharma Co., Japan). Endometrial preparation for frozen-thawed embryo transfer cycles was conducted using either natural cycles or artificial cycles. For patients with regular menstrual cycles and normal ovulation, a natural cycle protocol was adopted. Transvaginal ultrasound monitoring of follicular development and endometrial status was initiated on days 8–10 of the menstrual cycle. Beginning on the first day after ovulation, oral dydrogesterone tablets (Duphaston, Abbott Laboratories, USA, 10 mg/tablet) were administered at 10 mg twice daily (bid). For patients with irregular menstrual cycles or ovulatory dysfunction, a hormone replacement cycle protocol was implemented. Oral estradiol valerate tablets (Progynova, Bayer AG, Germany, 1 mg/tablet) were administered starting on day 2 of the menstrual cycle at 2 mg bid or 3 mg bid. After 10 days of treatment, when endometrial thickness reached ≥7 mm as confirmed by ultrasound monitoring, oral dydrogesterone (10 mg bid) and intravaginal progesterone gel (Crinone, Merck Serono, Switzerland, 90 mg/tube) at 90 mg once daily (qd) were added to initiate endometrial transformation.

#### Pregnancy outcomes and observation indicators

Biochemical pregnancy was defined as a positive serum human chorionic gonadotropin (hcg) test result detected 14 days after embryo transfer, indicating initial embryonic implantation without ultrasonographic evidence of pregnancy; clinical pregnancy was confirmed by transvaginal ultrasound examination performed 28–30 days after embryo transfer, which identified the presence of an intrauterine gestational sac combined with a visible embryonic heartbeat; miscarriage was defined as spontaneous termination of a confirmed clinical pregnancy with spontaneous loss of the embryo or fetus before 28 weeks of gestational age; live birth was defined as the delivery of a fetus with demonstrable vital signs, including spontaneous breathing, heartbeat, and umbilical cord pulsation, at a gestational age of ≥28 weeks, regardless of whether the neonate survived after delivery.

All primary and secondary outcomes were calculated on a per-patient basis. CLBR was defined as the cumulative probability of live birth from the first oocyte retrieval until live birth occurred or all usable embryos were used up. The control group received treatment within a single oocyte retrieval cycle. The embryo accumulation group required multiple consecutive oocyte retrieval cycles to collect embryos prior to frozen-thawed transfer, and its follow-up covered the entire multi-cycle treatment process.

Available embryo rate = Number of available embryos/Number of normal 2PN cleavage embryos × 100%; High-quality embryo rate = Number of high-quality embryos/Number of normal 2PN cleavage embryos × 100%; Cumulative clinical pregnancy rate = Number of patients achieving the first clinical pregnancy after initial oocyte retrieval/Total number of included oocyte retrieval cycles × 100%; Miscarriage rate = Number of miscarriage cycles/Number of clinical pregnancy cycles × 100%; Cumulative live birth rate = Number of patients achieving the first live birth after initial oocyte retrieval/Total number of included oocyte retrieval cycles × 100%.

### Statistical analyses

All statistical analyses were performed using SPSS 29.0 and R 4.x. The normality of continuous data was tested using the Kolmogorov-Smirnov test. Normally distributed data were presented as the mean ± standard deviation and compared by the independent t-test, while non-normally distributed data were expressed as the median (interquartile range) and analyzed using the Mann-Whitney U test. Categorical variables were summarized as percentages and compared using the Chi-square test or Fisher’s exact test. To minimize baseline confounding bias between groups, 1:1 propensity score matching (PSM) was performed. Baseline balance after matching was evaluated using the standardized mean difference (SMD), and |SMD| < 0.1 was considered indicative of adequate balance. Multivariable logistic regression models with hierarchical covariate adjustment were used to explore the association between cumulative embryo number and pregnancy outcomes in the full cohort, and survey-weighted logistic regression in the PSM-matched cohort was adopted for sensitivity analysis. Restricted cubic spline and threshold effect analyses were further conducted in the embryo accumulation group to characterize the nonlinear relationship and identify the optimal threshold of cumulative embryo number. All statistical tests were two-tailed, and P < 0.05 was considered statistically significant.

## Results

**Figure 1:**
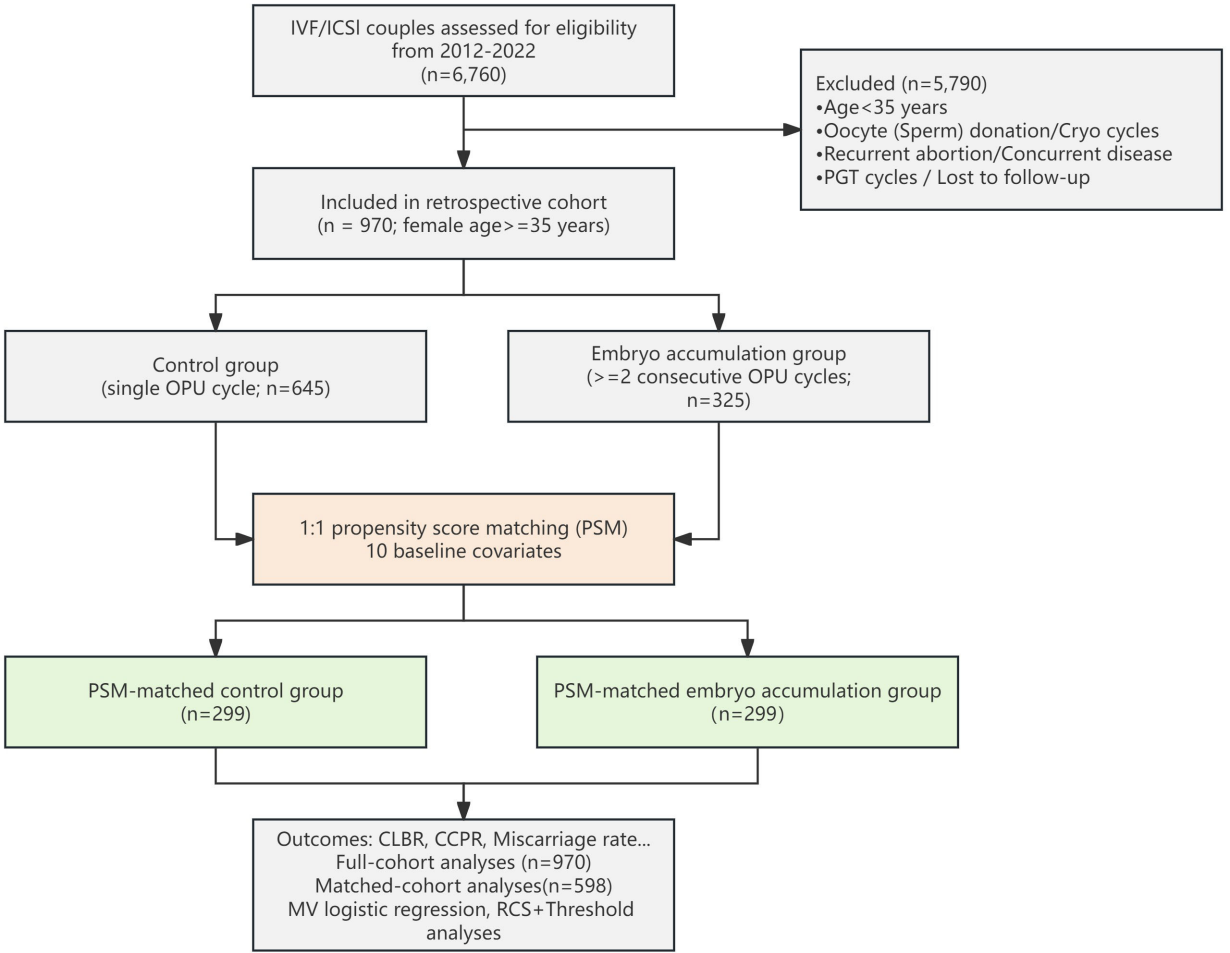
Flowchart depicting IVF/ICSI cohort selection from 2012 to 2022, exclusion criteria, division into control and embryo accumulation groups, 1:1 propensity score matching on ten baseline covariates, final matched groups, and outcome analyses including CLBR, CCPR, and miscarriage rate. CLBR, cumulative live birth rate; CCPR, cumulative clinical pregnancy rate.

A total of 6760 couples were initially screened, and 970 women met the inclusion criteria. Among them, 325 patients received the embryo accumulation strategy, whereas the remaining 645 patients underwent conventional treatment without embryo accumulation. After 1:1 propensity score matching of baseline characteristics, 299 cycles were finally enrolled in both the embryo accumulation group and the control group (**Figure 1**).

Before PSM, significant differences in baseline characteristics were identified between the embryo accumulation group and the control group. Patients in the embryo accumulation group exhibited significantly higher maternal and paternal ages, elevated basal follicle-stimulating hormone levels, and a higher proportion of diminished ovarian reserve, but lower anti-Müllerian hormone levels, and thinner endometrial thickness on embryo transfer day in the first oocyte retrieval cycle compared with the control group (all P < 0.05). After PSM, the absolute values of |SMD| for all baseline variables were less than or close to 0.1, suggesting that the two groups achieved adequate baseline balance and possessed good intergroup comparability (**Table 1**).

**Table 1:** Comparison of baseline characteristics between the embryo accumulation group and the control group.

Characteristic	Before PSM				After PSM				
	Embryo accumulation group (n = 325)	Control group (n = 645)	χ²/t/Z	P value	Embryo accumulation group (n = 299)	Control group (n = 299)	χ²/t/Z	P value	|SMD|
Female age, years	39.0(37.0, 42.0)	38.0(36.0, 40.0)	5.758	<0.001	39.0(37.0, 42.0)	39.0(37.0, 42.0)	0.226	0.821	0.004
Male age, years	39.0(36.0, 43.0)	38.0(36.0, 41.0)	3.339	<0.001	39.0(37.0, 43.0)	39.0(36.0, 42.0)	0.444	0.656	0.018
BMI, kg/m2	23.3(21.1, 26.0)	23.4(21.2, 25.8)	-0.402	0.688	23.4(21.1, 26.1)	23.4(21.4, 25.9)	-0.014	0.989	0.016
Duration of infertility, years	4.0(2.0, 8.0)	4.0(2.0, 8.4)	-0.400	0.689	4.0(2.0, 8.0)	4.0(2.0, 8.6)	0.457	0.648	0.009
Basal FSH, IU/mL	9.8(7.8, 14.2)	8.6(6.9, 11.2)	5.018	<0.001	9.9(7.7, 14.3)	9.6(7.3, 12.8)	1.836	0.066	0.148
Basal LH, IU/mL	3.9(2.9, 5.4)	3.8(2.8, 5.2)	1.172	0.241	3.9(2.9, 5.4)	3.9(2.9, 5.2)	0.810	0.418	0.038
Basal E2, pg/mL	41.0(27.0, 65.0)	42.0(28.0, 63.5)	-0.779	0.436	41.0(27.0, 64.5)	45.5(31.0, 73.0)	-2.289	0.022	0.17
AMH, ng/mL	0.6(0.2, 1.2)	1.2(0.5, 2.2)	-8.522	<0.001	0.6(0.2, 1.2)	0.8(0.3, 1.4)	-2.354	0.019	0.184
AFC	7.0(3.0, 11.0)	7.0(3.0, 11.0)	-1.109	0.266	7.0(3.0, 11.0)	6.0(3.0, 11.0)	0.193	0.846	0.013
Infertility type - Primary	28.92% (94/325)	29.92% (193/645)	0.061	0.805	28.09% (84/299)	27.76% (83/299)	0.000	1.000	
Infertility type - Secondary	71.08% (231/325)	70.08% (452/645)	0.061	0.805	71.91% (215/299)	72.24% (216/299)	0.000	1.000	
Tubal factor (%)	56.31% (183/325)	56.59% (365/645)	0.000	0.988	57.86% (173/299)	54.52% (163/299)	0.550	0.458	
Ovulatory disorder (%)	1.54% (5/325)	2.95% (19/645)	1.238	0.266	1.34% (4/299)	1.34% (4/299)	Fisher	1.000	
Endometrial lesion EM (%)	7.38% (24/325)	7.91% (51/645)	0.026	0.873	8.03% (24/299)	9.03% (27/299)	0.086	0.770	
Uterine factor (%)	7.08% (23/325)	6.98% (45/645)	0.000	1.000	7.36% (22/299)	10.03% (30/299)	1.032	0.310	
DOR (%)	26.46% (86/325)	17.36% (112/645)	10.456	0.001	25.42% (76/299)	23.08% (69/299)	0.328	0.567	
Male factors (%)	0.92% (3/325)	2.02% (13/645)	0.988	0.320	1.00% (3/299)	1.34% (4/299)	Fisher	1.000	
Endometrial thickness (mm)	9.0(8.0, 11.0)	10.0(8.4, 11.0)	-3.129	0.002	9.0(8.0, 11.0)	10.0(8.0, 11.0)	-1.362	0.170	0.095
Fertilization method - IVF	69.85% (227/325)	68.99% (445/645)	0.039	0.843	70.23% (210/299)	72.24% (216/299)	0.204	0.651	
Fertilization method - ICSI	30.15% (98/325)	31.01% (200/645)	0.039	0.843	29.77% (89/299)	27.76% (83/299)	0.204	0.651	

### Ovulation stimulation and embryo laboratory indicators

The embryo accumulation group had significantly lower total gonadotropin (Gn) dosage and shorter Gn stimulation duration than the control group; the differences were statistically significant (P < 0.05). The embryo accumulation group presented higher counts of oocyte retrieval cycles, embryo transfer cycles, cumulative embryos, and transferred embryos; the differences were statistically significant (P < 0.05). By contrast, the number of oocytes retrieved, oocytes retrieved per cycle, and available embryos per cycle were significantly lower in the embryo accumulation group (P < 0.05). No significant intergroup differences were found in the number of available embryos, number of high-quality embryos, high-quality embryo rate and embryo developmental days. (P > 0.05). Additionally, the control group exhibited a significantly higher proportion of fresh cycles than the embryo accumulation group (P < 0.05) (**Table 2**).

**Table 2:** Comparison of ovulation stimulation and laboratory indicators between the embryo accumulation group and the control group.

Items	Before PSM				After PSM				
	Embryo accumulation group (n = 325)	Control group (n = 645)	χ²/t/Z	P value	Embryo accumulation group (n = 299)	Control group (n = 299)	χ²/t/Z	P value	|SMD|
Gn starting dose (IU)	300.0(225.0, 300.0)	300.0(225.0, 300.0)	-1.263	0.180	300.0(225.0, 300.0)	300.0(225.0, 300.0)	-1.777	0.058	0.245
Total Gn dose (IU)	2343.8(1575.0, 3000.0)	2625.0(2025.0, 3300.0)	-4.781	<0.001	2400.0(1575.0, 3000.0)	2550.0(2025.0, 3381.2)	-3.758	<0.001	0.363
Gn duration (days)	9.0(7.0, 10.0)	9.0(8.0, 11.0)	-5.274	<0.001	9.0(7.0, 10.0)	9.0(8.0, 11.0)	-3.925	<0.001	0.372
Oocyte retrieval cycles	3.0(2.0, 4.0)	1.0(1.0, 2.0)	19.387	<0.001	2.0(2.0, 4.0)	1.0(1.0, 2.0)	14.920	<0.001	1.021
Embryo transfer cycles	2.0(1.0, 2.0)	1.0(1.0, 2.0)	5.618	<0.001	2.0(1.0, 2.0)	1.0(1.0, 2.0)	3.380	<0.001	0.242
Cumulative embryo count	3.0(2.0, 4.0)	1.0(1.0, 2.0)	13.568	<0.001	3.0(2.0, 4.0)	1.0(1.0, 3.0)	9.536	<0.001	0.581
Embryos transferred	2.0(1.0, 2.0)	1.0(1.0, 1.0)	8.650	<0.001	2.0(2.0, 2.0)	1.0(1.0, 2.0)	6.589	<0.001	0.802
Oocytes retrieved	2.0(1.0, 4.0)	3.0(2.0, 5.0)	-5.758	<0.001	2.0(1.0, 4.0)	3.0(1.0, 5.0)	-3.087	0.002	0.259
Oocytes retrieved per single cycle	0.7(0.3, 1.5)	2.0(1.0, 4.0)	-15.525	<0.001	0.7(0.3, 1.5)	2.0(1.0, 3.0)	-11.338	<0.001	0.769
Available embryo	1.0(1.0, 2.0)	1.0(1.0, 1.0)	2.588	0.001	1.0(1.0, 2.0)	1.0(1.0, 2.0)	0.932	0.269	0.04
Available embryos per single cycle	0.5(0.3, 0.7)	1.0(1.0, 1.0)	-15.652	<0.001	0.5(0.3, 0.7)	1.0(1.0, 1.0)	-12.843	<0.001	0.984
High-quality embryo	75.0(50.0, 100.0)	100.0(0.0, 100.0)	1.385	0.130	100.0(50.0, 100.0)	100.0(0.0, 100.0)	0.439	0.632	0.082
High-quality embryo rate (%)	65.65% (279/425)	57.80% (437/756)	6.686	0.010	66.33% (262/395)	61.94% (223/360)	1.391	0.238	
Embryo stage transferred - Cleavage	93.70% (238/254)	94.58% (576/609)	0.121	0.728	94.02% (220/234)	90.94% (251/276)	1.288	0.256	
Embryo stage transferred - Blastocyst	8.27% (21/254)	5.42% (33/609)	2.018	0.155	8.12% (19/234)	9.06% (25/276)	0.047	0.828	0.033
Cycle type - fresh	45.28% (120/265)	83.52% (532/637)	134.648	<0.001	44.49% (109/245)	71.48% (208/291)	38.983	<0.001	
Cycle type - FET	53.58% (142/265)	16.48% (105/637)	127.690	<0.001	54.29% (133/245)	28.52% (83/291)	35.634	<0.001	

### Pregnancy outcomes indicators

After PSM, the embryo accumulation group remained significantly higher biochemical pregnancy rate, cumulative clinical pregnancy rate, and cumulative live birth rate than the control group, the differences were statistically significant (P < 0.001). No significant intergroup difference was observed in the miscarriage rate (P > 0.05). Additionally, the embryo accumulation group showed a significantly higher cycle cancellation rate and a longer interval from initial oocyte retrieval to delivery (all P < 0.001), while the multiple pregnancy rate was comparable between the two groups with no significant difference (P > 0.05) (**Table 3**). Kaplan–Meier survival analysis with a 12-month fixed follow-up anchored at the time of initial oocyte retrieval was performed to assess the time-to-live-birth outcome, with relevant results detailed in **Supplementary Tables 2**, **3**.

**Table 3:** Comparison of pregnancy outcome indicators between the embryo accumulation group and the control group after PSM.

Items	Embryo accumulation group (n = 299)	Control group (n = 299)	χ²/t/Z	P value
Cycle cancellation rate (%)	31.77% (95/299)	13.38% (40/299)	27.898	<0.001
Singleton delivery rate (%)	70.11% (61/87)	79.31% (46/58)	1.083	0.298
Twin delivery rate (%)	21.84% (19/87)	12.07% (7/58)	1.642	0.200
Duration from first oocyte retrieval to delivery (months), median (IQR)	10.6(8.4, 14.3)	8.6(8.4, 9.5)	3.102	0.002
Biochemical pregnancy rate (%)	45.48% (136/299)	29.43% (88/299)	15.768	<0.001
Cumulative clinical pregnancy rate (%)	43.81% (131/299)	28.76% (86/299)	14.003	<0.001
Cumulative live birth rate (%)	37.79% (113/299)	23.41% (70/299)	13.890	<0.001
Miscarriage rate (%)	13.74% (18/131)	17.44% (15/86)	0.302	0.583

### Results of logistic regression and threshold effect analysis

**Figure 2:**
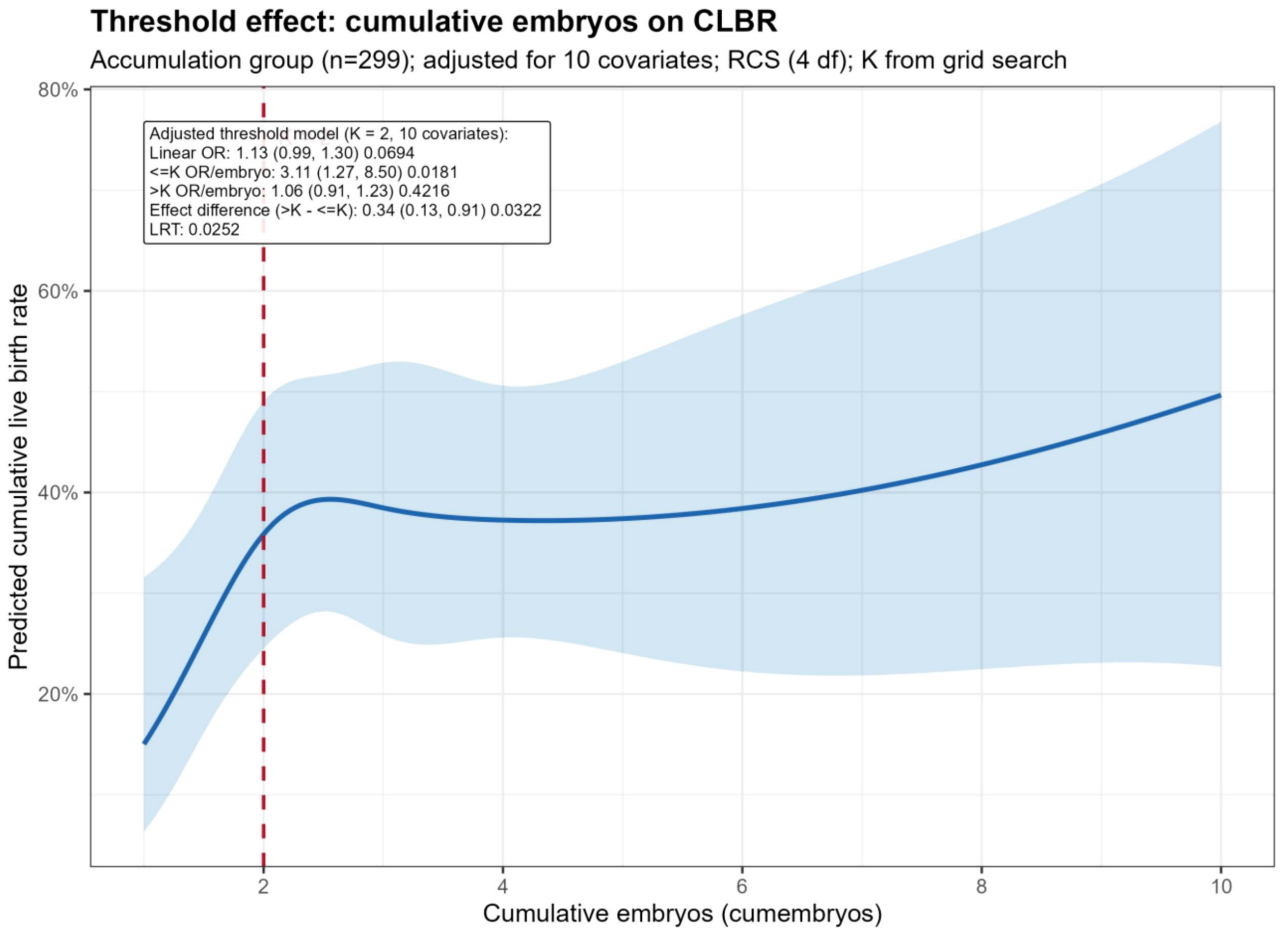
Line chart shows predicted cumulative live birth rate increasing with the number of cumulative embryos; vertical dashed red line at 2 embryos indicates a key threshold; shaded blue area shows confidence interval; boxed legend details statistical model parameters and significance levels.

**Figure 3:**
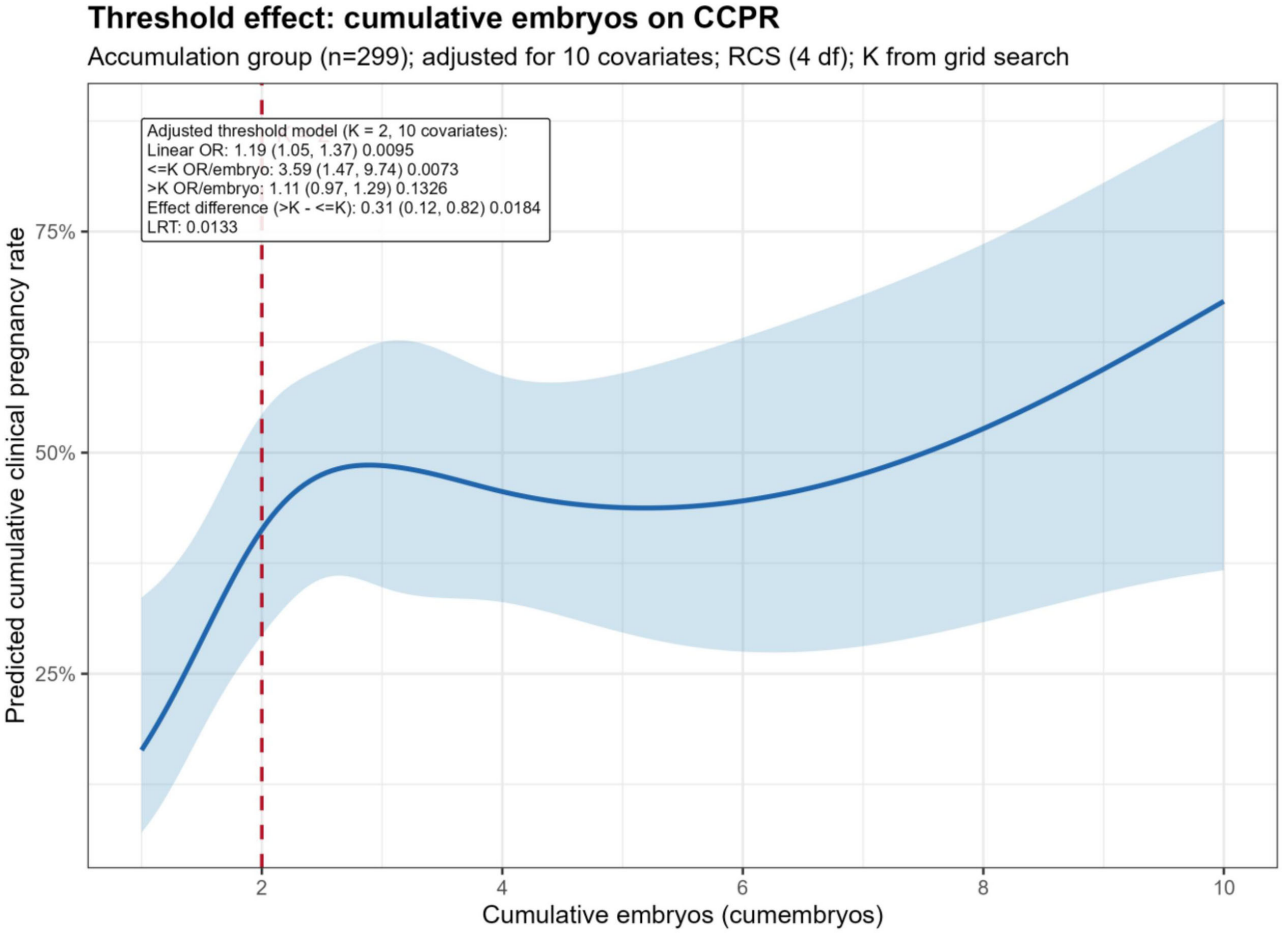
Line graph shows predicted cumulative clinical pregnancy rate versus cumulative embryos, with a threshold at two embryos marked by a red dashed line. The blue line indicates a nonlinear trend, with a shaded area showing confidence intervals. Model results in a textbox note a significant threshold effect and provides odds ratios and p-values for thresholds and linear components.

Multivariable logistic regression analysis based on the fully adjusted model (Adjust II) showed that embryo accumulation was independently correlated with CLBR and CCPR. Embryo accumulation yielded a significant improvement in CLBR (OR = 2.75, 95% CI: 1.87–4.05, P < 0.001) and CCPR (OR = 2.71, 95% CI: 1.87–3.92, P < 0.001) (**Tables 4**, **5**). Threshold effect analysis was performed in the embryo accumulation group, and two embryos were identified as the cut-off value. When the cumulative embryo number was ≤ 2, significant correlations were observed between cumulative embryo number and both CLBR (OR = 3.11, 95% CI: 1.27–8.50, P = 0.0181) and CCPR (OR = 3.59, 95% CI: 1.47–9.74, P = 0.0073). When the cumulative embryo number exceeded this threshold, the beneficial effect plateaued. No significant associations were detected for CLBR (OR = 1.06, 95% CI: 0.91–1.23, P = 0.4216) and CCPR (OR = 1.11, 95% CI: 0.97–1.29, P = 0.1326) (**Tables 6**, **7**, **Figures 2**, **3**).

**Table 4:** The effect of embryo accumulation group on cumulative clinical live birth rate.

Exposure	Non-adjusted	Adjust I	Adjust II
Group			
Control group	1	1	1
Embryo accumulation group	1.67 (1.26, 2.22) 0.0004	2.32 (1.68, 3.22) 0.00000039	2.75 (1.87, 4.05) 0.00000028

Data presented: OR (95% CI) P value.

Outcome: cumulative clinical live birth rate (CLBR per person).

Reference: Control group (OR = 1).

Analysis: full cohort; unweighted binary logistic regression.

Non-adjusted: exposure only.

Adjust I: female age, male age, BMI, AMH.

Adjust II: 10 baseline covariates used in propensity score matching (female age, male age, BMI, bFSH, AFC, AMH, infertility factors, infertility duration, infertility type, and cycle type).

**Table 5:** The effect of embryo accumulation group on cumulative clinical pregnancy rate.

Exposure	Non-adjusted	Adjust I	Adjust II
Group			
Control group	1	1	1
Embryo accumulation group	1.62 (1.23, 2.13) 0.0006	2.19 (1.60, 2.99) 0.00000079	2.71 (1.87, 3.92) 0.00000015

Data presented: OR (95% CI) P value.

Outcome: cumulative clinical pregnancy rate (CCPR per person).

Reference: Control group (OR = 1).

Analysis: full cohort; unweighted binary logistic regression.

Non-adjusted: exposure only.

Adjust I: female age, male age, BMI, AMH.

Adjust II: 10 baseline covariates used in propensity score matching (female age, male age, BMI, bFSH, AFC, AMH, infertility factors, infertility duration, infertility type, and cycle type).

**Table 6:** Threshold effect analysis for cumulative embryos of impact on cumulative live birth rate.

Outcome	Cumulative live birth rate (CLBR per person)
Model I | One-line effect	1.13 (0.99, 1.30) 0.0694
Model II | Turning point (K)	2
Model II | < K effect 1	3.11 (1.27, 8.50) 0.0181
Model II | > K effect 2	1.06 (0.91, 1.23) 0.4216
Model II | effect2 - 1	0.34 (0.13, 0.91) 0.0322
Model fit value at K	-0.60 (-1.07, -0.12)
LRT test	0.0252

Data: OR (95% CI) P value/beta (95% CI) at K.

Outcome: cumulative live birth rate (CLBR per person). Exposure: cumulative embryos (continuous).

Turning point K selected by maximum log-likelihood among K = 2–7 (segment n ≥ 10).

Adjusted for female age, male age, BMI, bFSH, AFC, AMH, infertility factors, infertility duration, infertility type, and cycle type.

**Table 7:** Threshold effect analysis for cumulative embryos of impact on cumulative clinical pregnancy rate.

Outcome	Cumulative clinical pregnancy rate (CCPR per person)
Model I | One-line effect	1.19 (1.05, 1.37) 0.0095
Model II | Turning point (K)	2
Model II | < K effect 1	3.59 (1.47, 9.74) 0.0073
Model II | > K effect 2	1.11 (0.97, 1.29) 0.1326
Model II | effect2 - 1	0.31 (0.12, 0.82) 0.0184
Model fit value at K	-0.35 (-0.81, 0.11)
LRT test	0.0133

Data: OR (95% CI) P value/beta (95% CI) at K.

Outcome: cumulative live birth rate (CLBR per person). Exposure: cumulative embryos (continuous).

Turning point K selected by maximum log-likelihood among K = 2–7 (segment n ≥ 10).

Adjusted for female age, male age, BMI, bFSH, AFC, AMH, infertility factors, infertility duration, infertility type, and cycle type.

## Discussion

Advanced maternal age is a well-recognized independent determinant of adverse IVF/ICSI prognosis, predominantly characterized by progressive ovarian reserve attrition, diminished per-cycle oocyte and embryo yields, and deteriorated embryo quality. These aging-related alterations collectively contribute to reduced cumulative pregnancy and live birth rates in women of advanced reproductive age. Effectively improving assisted reproductive outcomes in this population remains a challenging and highly debated topic in reproductive medicine. This retrospective cohort study enrolled women aged ≥35 years undergoing IVF/ICSI treatment. Following propensity score matching, consecutive-cycle embryo accumulation was significantly associated with elevated CLBR and CCPR. Fully adjusted multivariable regression analysis further verified that embryo accumulation acts as an independent protective factor for favorable reproductive outcomes, with superior clinical efficacy observed in patients with no more than two accumulated embryos.

The progressive decline in primordial follicle count leads to an exponential reduction in ovarian reserve in women after the age of 37 (18). As the total remaining follicle pool diminishes, the number of available follicles decreases correspondingly, resulting in fewer available embryos and ultimately contributing to poor ART outcomes in advanced maternal age. In this study, compared with the control group, the accumulation of embryos group exhibited significantly higher maternal and paternal ages, baseline FSH levels, and proportion of DOR, while demonstrating lower AMH levels, and endometrial thickness on the day of embryo transfer (P < 0.05). These findings indicate that patients in the embryo accumulation group were characterized by advanced age, poor ovarian reserve, and suboptimal endometrial receptivity, all of which contribute to reduced oocyte yield, insufficient available embryos, and decreased implantation rates in women of advanced reproductive age. To compensate for single-cycle therapeutic limitations, repeated ovarian stimulation combined with embryo accumulation can increase the total number of oocyte retrieval cycles, embryo transfer cycles, and cumulative transferred embryos. The present study showed that the embryo accumulation group had lower oocyte yield and fewer usable embryos per cycle. Even so, this regimen achieved markedly higher CCPR and CLBR than conventional single-cycle treatment (P < 0.001). Considering the substantial influence of maternal age on reproductive outcomes, we further conducted age-stratified subgroup analyses to compare CCPR and CLBR between the two groups. For CLBR, the rates were 59.41% versus 37.17% in patients aged 35–37 years, 38.54% versus 26.06% in those aged 38–40 years, 25.00% versus 9.52% in those aged 41–43 years, and 12.5% versus 0 in women over 43 years. For CCPR, the corresponding rates were 62.38% versus 43.09%, 42.71% versus 32.45%, 33.75% versus 14.29%, and 20.83% versus 0 (**Supplementary Table 1**). Sensitivity analyses were additionally performed to verify the robustness of the above findings (**Supplementary Table 7**). Collectively, these results confirm that embryo accumulation can effectively improve reproductive outcomes for women of advanced reproductive age, which is consistent with the conclusions reported in multiple previous studies (12, 19–22). In the study by CoAb et al. (12), the CLBR in poor responders of the cumulative oocyte group reached approximately 55% after two embryo transfer cycles, demonstrating a significant increase in CLBR and confirming the efficacy of this approach in the management of poor responders. Other scholars (19–22) have investigated CLBR in populations classified by the Bologna criteria or the POSEIDON criteria. Their studies demonstrated that women with poorer prognoses exhibited lower CLBR after one treatment cycle but achieved better outcomes after three IVF/ICSI cycles. Across POSEIDON groups 1, 2, 3, 4, CLBR showed progressive increases from the first to the third treatment cycle, indicating that despite their infertile status, women with low prognosis could enhance their conception probability through multiple IVF/ICSI cycles.

However, some studies have reported conclusions contradictory to ours, suggesting that the multiple stimulation cycles with embryo cryopreservation strategy does not improve CLBR in poor ovarian responders, but rather prolongs the time to achieve live birth (23). This discrepancy may be rationally interpreted based on the distinct study populations and embryo accumulation criteria in the present study. First, previous contradictory studies strictly enrolled patients diagnosed with POR according to the Bologna criteria, who presented extremely severe ovarian dysfunction with highly compromised oocyte quantity and quality. In comparison, our study included women aged ≥35 years with heterogeneous ovarian reserve, representing a broader and less severely impaired clinical cohort. Second, those studies required the accumulation of at least five embryos before transfer, which necessitated repeated treatment cycles and substantially prolonged the time to final embryo transfer and live birth. In contrast, the present threshold effect analysis confirmed that favorable reproductive outcomes could be maximized with no more than two accumulated embryos in advanced-age populations. This low-threshold accumulation strategy avoids excessive repeated stimulation cycles, thereby effectively balancing treatment duration and reproductive benefits.

Consistent with the viewpoints of previous literature, our study also confirmed that the embryo accumulation strategy inevitably prolongs the interval from initial oocyte retrieval to live birth after PSM matching. Such a time delay is clinically reasonable and inherent to the multi-cycle accumulation protocol, as repeated stimulation and embryo cryopreservation are required to obtain sufficient cryopreserved embryos for subsequent frozen embryo transfer. Furthermore, stratified follow-up analysis with a unified 12-month clinical endpoint showed that the control group exhibited a higher short-term CLBR within 12 months, while the embryo accumulation group required a longer treatment duration to achieve sustained reproductive outcomes. However, longitudinal follow-up analyses indicated that the embryo accumulation group presented higher cumulative live birth rates than the control group starting at 18 months of follow-up, and this intergroup discrepancy continued to expand during the 24- to 36-month follow-up phase (**Supplementary Table S3a, b**). Accordingly, the major clinical strength of the embryo accumulation strategy lies in its favorable long-term cumulative prognosis. In contrast to the high embryo accumulation criteria adopted in prior studies, the present analysis suggested that optimal clinical efficacy could be achieved with a relatively low cumulative embryo threshold (no more than two embryos) among advanced-age patients. This mild accumulation strategy can reduce redundant treatment cycles induced by excessive embryo accumulation and help elevate cumulative live birth rates in this population. Advanced maternal age not only reduces the quantity of oocytes and available embryos but also significantly compromises their quality. In women aged over 35 years, meiotic chromosome segregation errors become increasingly prevalent, resulting in aneuploid oocytes. When fertilized by sperm, these aneuploid oocytes develop into aneuploid embryos, which are predisposed to spontaneous abortion. Furthermore, Advancing female age is associated with elevated risks of both infertility and pregnancy loss (24). Patients requiring massive embryo accumulation are generally older, and the age-related deterioration in embryonic quality cannot be compensated merely by increasing embryonic quantity. Therefore, unlimited embryo accumulation fails to improve and may even offset cumulative reproductive benefits, whereas individualized low-threshold embryo accumulation achieves a better balance between treatment timeline and long-term pregnancy outcomes.

This study further conducted smooth curve fitting and threshold effect analysis to explore the optimal cumulative embryo threshold for advanced-age patients undergoing IVF/ICSI. The analyses demonstrated that the clinical benefits of embryo accumulation exhibited a threshold effect, with two embryos identified as the optimal cumulative cutoff value for this population. Specifically, increased cumulative embryo quantity within the threshold of two embryos was significantly associated with elevated CLBR and CCPR, whereas further embryo accumulation yielded plateaued therapeutic effects without additional improvements in long-term reproductive prognosis, indicating that excessive embryo accumulation cannot further optimize ART outcomes in advanced-age women. Age-stratified threshold analyses were subsequently performed to explore the differential threshold characteristics across various maternal age subgroups. The results showed a significant nonlinear correlation between cumulative embryo number and reproductive outcomes in patients aged 38–40 years, as evidenced by significant likelihood ratio test results. However, no stable threshold turning point (K value) was obtained in this subgroup, which was likely caused by limited sample size, scattered data distribution, and unstable segmented regression coefficients. By contrast, no valid threshold values or significant nonlinear trends were observed in the 35–37 years and ≥41 years subgroups. Such inconsistent analytical findings across age subgroups indicate the presence of heterogeneity in the threshold effect of embryo accumulation among advanced-age subgroups (**Supplementary Tables 4**, **5**). The plateaued therapeutic benefits of excessive embryo accumulation are primarily attributed to inter-individual differences in age distribution, as patients requiring more embryos for accumulation tend to be older and possess inferior reproductive potential. Advanced maternal age is a crucial independent factor affecting ART prognosis. with pregnancy and live birth rates decreasing progressively with age, particularly in women over 40 years of age. Existing epidemiological data and clinical studies have validated this age-related decline in reproductive capacity. Specifically, 2020 epidemiological statistics from the Centers for Disease Control and Prevention (CDC) and the Society for Assisted Reproductive Technology (SART) indicated a progressive decline in CLBR with advancing maternal age, with a CLBR of 52.0% in women younger than 35 years, 38.1% in those aged 35–37 years, 23.5% in those aged 38–40 years, 11.2% in those aged 41–42 years, and merely 3.2% in women over 43 years (25). A domestic retrospective study involving 4102 advanced-age IVF/ICSI patients also verified the progressive reduction in per-cycle LBR as maternal age increases (26). Multiple ART cycles can compensate for inadequate single-cycle embryo yield and improve cumulative reproductive outcomes in patients younger than 43 years, with the 40–42- years subgroup achieving a maximum LBR of 40.3% after four treatment cycles. However, patients aged 43 years and above exhibit poor per-cycle reproductive performance, with a CLBR of only 5.5% even after four cycles of embryo accumulation. Consistent with previous findings, the present study confirms that multi-cycle embryo accumulation benefits advanced-age patients, while its efficacy is constrained by age-related reproductive decline. Increased embryo quantity cannot compensate for the deterioration in autologous oocyte and embryonic quality associated with aging. Yuan et al. (19) reported that CLBR improves within the first three stimulation cycles; yet, the incremental clinical benefit weakens and plateaus in subsequent cycles due to gradual antral follicle depletion, which supports the plateaued therapeutic effect of excessive embryo accumulation observed in this study. Combined with the heterogeneous threshold effects among age subgroups, these findings indicate that simply increasing embryo accumulation fails to yield sustained improvements in ART outcomes for advanced-age patients. Therefore, individualized low-threshold embryo accumulation strategies tailored to maternal age are recommended to optimize clinical efficacy and reduce unnecessary treatment burden.

With rigorous screening criteria and multiple statistical approaches, including propensity score matching, multivariate logistic regression, and sensitivity analysis, the present study ensured stable and credible analytical results. It also preliminarily explored the optimal cumulative embryo threshold and age-subgroup heterogeneity for advanced-age patients, providing preliminary evidence for individualized embryo accumulation regimens. Nevertheless, several important limitations inherent to this retrospective study deserve discussion. First, this was a single-center retrospective study with a constrained sample size after strict case screening. Such sample and study design limitations may compromise the stability of threshold effect modeling, which contributes to the failure to identify stable threshold turning points and fully clarify the heterogeneous threshold effects across different advanced-age subgroups. Future large-sample, multicenter randomized controlled trials with refined age stratification are needed to further verify and consolidate the findings and establish differentiated embryo accumulation schemes for precise individualized ART clinical decision-making. Second, residual unmeasured confounding factors could not be entirely excluded in this retrospective analysis. Specifically, patients capable of completing multi-cycle embryo accumulation generally exhibited better physical tolerance, economic conditions, and treatment compliance. These individualized clinical and socioeconomic indicators cannot be fully quantified and adjusted in statistical models, which may introduce inherent selection bias and interfere with the true clinical effect of embryo accumulation on reproductive outcomes. Third, the present study mainly analyzed clinical pregnancy and live birth outcomes. Relevant data regarding treatment costs, patient psychological status, and treatment tolerability were unavailable and could not be collected in this retrospective cohort. Therefore, this study cannot fully reflect the comprehensive clinical value and patient-reported outcomes of multi-cycle accumulation strategies. However, Datta AK et al. (17) argue that the benefits of adopting a cumulative embryo transfer strategy over three IVF/ICSI cycles outweigh the foreseeable risks. Future analyses should comprehensively account for multiple decision-influencing factors, including the patient’s physical condition, psychological state, and economic costs, to derive more robust conclusions.

## Conclusions

This retrospective cohort study investigated the impact of sequential embryo accumulation on IVF/ICSI outcomes in women of advanced reproductive age. Embryo accumulation independently enhanced cumulative clinical pregnancy and live birth rates in this population, with maximal clinical benefits achieved at a cumulative embryo number of ≤2. Additional embryo accumulation did not yield substantial improvements in long-term reproductive prognosis. Although multi-cycle embryo accumulation prolonged treatment duration and presented inferior short-term outcomes compared with single-cycle treatment, it effectively compensated for insufficient single-cycle embryo yield and improved the adverse reproductive outcomes associated with advanced age. The threshold effect of cumulative embryos varied across different age subgroups, suggesting heterogeneous optimal accumulation strategies among advanced-age patients. Given the inherent limitations of retrospective analysis and potential residual confounding factors, individualized treatment protocols integrating patient age, ovarian reserve, and treatment tolerance are recommended to optimize reproductive benefits and minimize treatment burden for advanced-age women undergoing ART.

## Data Availability

The original contributions presented in the study are included in the article/**Supplementary Material**. Further inquiries can be directed to the corresponding author.
