# Supplementary material for: Accumulation of embryos to improve outcomes in advanced-age women undergoing IVF/ICSI cycles: a retrospective cohort study
Source: Front Endocrinol (Lausanne). 2026 Jul 7;17:1859978. doi: 10.3389/fendo.2026.1859978 (PMC13385494; doi:10.3389/fendo.2026.1859978)
Supplement: Supplementary file 1 [file Table1.docx]

**Supplementary Materials (Revised)**

**Table S1. Age-stratified CLBR and CCPR (full cohort)**

| **Age group (years)** | **Accumulation CLBR** | **Control CLBR** | **CLBR P value** | **Accumulation CCPR** | **Control CCPR** | **CCPR P value** |
| --- | --- | --- | --- | --- | --- | --- |
| 35-37 | 60 (59.41%) | 113 (37.17%) | <0.001 | 63 (62.38%) | 131 (43.09%) | 0.001 |
| 38-40 | 37 (38.54%) | 49 (26.06%) | 0.043 | 41 (42.71%) | 61 (32.45%) | 0.115 |
| 41-43 | 20 (25.00%) | 10 (9.52%) | 0.009 | 27 (33.75%) | 15 (14.29%) | 0.003 |
| >43 | 6 (12.50%) | 0 (0.00%) | 0.026 | 10 (20.83%) | 0 (0.00%) | 0.001 |

**Table S2. Time to cumulative live birth from index OPU**

| **Metric** | **Accumulation** | **Control** | **P-value** |
| --- | --- | --- | --- |
| Patients in the survival set | 294 | 298.0 | - |
| Live birth events (dated delivery) | 82 | 57.0 | - |
| Median time to live birth (months, events only) | 9.8 | 8.6 | - |
| Log-rank P (time to live birth from OPU) | - | - | 0.039 |
| Multivariate log-rank P | - | - | 0.039 |
| Cox HR (accumulation vs control) | 1.42 (1.02-2.00) | - | 0.040 |

PSM: 1:1 nearest neighbor, 10 covariates, 299 matched pairs (n = 598). The survival set excludes patients without (D0) oocyte retrieval date and REGCOD 2018080206. Events = live birth with recorded delivery date; non-events censored at 2022-12-31.

**Table S3a. Landmark cumulative live birth probabilities (Kaplan-Meier)**

| Cohort | Outcome | Accumulation group | Control group | P value |
| --- | --- | --- | --- | --- |
| Full cohort (n = 970) | 12-month CLBR | 16.62% | 23.72% | 0.014 |
| Full cohort (n = 970) | 12-month CCPR | 27.38% | 30.08% | 0.426 |
| OPU ≤ 2021-12-31† | 12-month CLBR | 15.86% | 23.38% | 0.010 |
| OPU ≤ 2021-12-31† | 12-month CCPR | 26.54% | 29.68% | 0.359 |

† Excludes 52 patients with oocyte pick-up in 2022 to ensure at least 12 months of follow-up potential.

**Table S3b. Landmark cumulative live birth probabilities (Kaplan-Meier)**

| **Months from (D0) OPU** | **Multi-cycle accumulation** | **Control** |
| --- | --- | --- |
| 6 | 0.34% | 0.00% |
| 12 | 16.80% | 18.38% |
| 18 | 25.28% | 19.47% |
| 24 | 26.78% | 19.47% |
| 36 | 28.81% | 19.92% |

Interpretation: Within the 12-month follow-up period, the control group showed a higher live birth rate. The higher CLBR in the cumulative group was mainly observed after 12 months of follow-up.

**Table S4. Age-stratified threshold effect on CLBR**

| **Age_Group** | **Model_Type** | **Turning_Point_K** | **Effect_leK** | **Effect_gtK** | **LRT_P** | **n** |
| --- | --- | --- | --- | --- | --- | --- |
| 35-37 | One-line Linear | — | 0.91 (0.71, 1.17); P=0.4713 | — | 0.3851 | 91 |
| 38-40 | One-line Linear | — | 1.59 (1.10, 2.31); P=0.0143 | — | <0.001 | 91 |
| ≥41 | One-line Linear | — | 1.15 (0.92, 1.43); P=0.2136 | — | 0.5768 | 117 |

Footnote: A turning point (K) is reported only for Piecewise Linear models (LRT P < 0.05 and stable segment ORs). One-line Linear with LRT not significant shows the LRT P-value. One-line Linear with "LRT sig.; unstable" indicates that the piecewise model fit better than linear at grid K but segment coefficients were unstable, so no K is reported; the ≤K column shows the linear overall OR per embryo.

Adjusted for male age, BMI, bFSH, AFC, AMH, infertility factors, infertility duration, and infertility type.

**Table S5. Age-stratified threshold effect on CCPR**

| **Age_Group** | **Model_Type** | **Turning_Point_K** | **Effect_leK** | **Effect_gtK** | **LRT_P** | **n** |
| --- | --- | --- | --- | --- | --- | --- |
| 35-37 | One-line Linear | — | 0.93 (0.72, 1.21); P=0.5939 | — | 0.2716 | 91 |
| 38-40 | One-line Linear | — | 1.79 (1.19, 2.70); P=0.0053 | — | <0.001 | 91 |
| ≥41 | One-line Linear | — | 1.21 (0.99, 1.48); P=0.0573 | — | 0.3028 | 117 |

Footnote: A turning point (K) is reported only for Piecewise Linear models (LRT P < 0.05 and stable segment ORs). One-line Linear with LRT not significant shows the LRT P-value. One-line Linear with "LRT sig.; unstable" indicates that the piecewise model fit better than linear at grid K but segment coefficients were unstable, so no K is reported; the ≤K column shows the linear overall OR per embryo.

Adjusted for male age, BMI, bFSH, AFC, AMH, infertility factors, infertility duration, and infertility type.

**Table S6. Fresh vs frozen-thaw cycles in the control group (per transfer)**

| **Outcome** | **Fresh cycle** | **Frozen-thaw cycle** | **P-value** |
| --- | --- | --- | --- |
| Transfer cycles, n | 532 | 105 | nan |
| Live birth per transfer | 126 (23.68%) | 24 (22.86%) | 0.955 |
| Clinical pregnancy per transfer | 155 (29.14%) | 34 (32.38%) | 0.583 |
| Miscarriage among clinical pregnancies | 27 (17.42%) | 9 (26.47%) | 0.329 |

**Table S7. Sensitivity analysis—PSM-matched survey-weighted logistic regression (10 covariates, n=598)**

| **Analysis** | **Outcome** | **N** | **Display** |
| --- | --- | --- | --- |
| PSM matched weighted logistic | CLBR | 598 | 2.51 (1.68, 3.75) <0.001 |
| PSM matched weighted logistic | CCPR | 598 | 2.44 (1.66, 3.59) <0.001 |

Adjusted for female age, male age, BMI, bFSH, AFC, AMH, infertility factors, infertility duration, infertility type, and cycle type.
